# A Non-Enzymatic Method to Obtain a Fat Tissue Derivative Highly Enriched in Adipose Stem Cells (ASCs) from Human Lipoaspirates: Preliminary Results

**DOI:** 10.3390/ijms19072061

**Published:** 2018-07-15

**Authors:** Francesco De Francesco, Silvia Mannucci, Giamaica Conti, Elena Dai Prè, Andrea Sbarbati, Michele Riccio

**Affiliations:** 1 Department of Reconstructive Surgery and Hand Surgery (SOD Chirurgia Ricostruttiva e Chirurgia della Mano), AOU “Ospedali Riuniti”, via Conca, 60126 Ancona, Italy; michele.riccio@ospedaliriuniti.marche.it; 2 Department of Neuroscience, Biomedicine and Movement, Human Anatomy and Histology Section, University of Verona, 37134 Verona, Italy; silman82@hotmail.com (S.M.); giamaica.conti@univr.it (G.C.); elena.daipre@univr.it (E.D.P.); andrea.sbarbati@univr.it (A.S.)

**Keywords:** adipose stem cells, adipose tissue, non-enzymatic method, Rigenera protocol, enzymatic digestion

## Abstract

Adipose tissue possesses phenotypic gene expression characteristics that are similar to human mesenchymal stem cells (hMSCs). Nevertheless, the multilineage potential may be inhibited, and cells may not expand adequately to satisfy the requirements of Good Manufacturing Practice (cGMP). An autologous hMSC-enriched fat product would fulfil the void from a biomedical and clinical perspective. In this study, we suggest a novel mechanism using a closed system without enzymes, additives or other modifications, which will produce non-expanded, accessible material. This decentralized fat product, unlike unprocessed lipoaspirates, adequately encloses the vascular stroma with adipocytes and stromal stalks along with their vascular channels and lumina. This fat product contained hASCs and fewer hematopoietic elements such as lipoaspirates, which were digested enzymatically according to flow cytometric investigations, and molecular analysis also showed significant hASC uniformity within the cells of the stromal vascular tissue. Moreover, the fat product produced a higher quantity of hASCs similar to hMSCs in isolation with the typical characteristics of an osteogenic, chondrogenic and adipogenic lineage. Interestingly, these properties were evident in the non-enzymatic derived adipose tissue, as opposed to hASCs in isolation from the enzymatically digested lipoaspirates, suggesting that the aforementioned procedure may be an adequate alternative to regenerate and engineer tissue for the treatment of various medical conditions and promote efficient patient recovery.

## 1. Introduction

Human mesenchymal stem cells (hMSCs) can renew themselves efficiently, grow rapidly and differentiate successfully, all of which make them excellent cell sources in regenerative medicine in different contexts [1]. Bone marrow can adequately produce hMSCs (hBMSCs) [2], but bone marrow collection is an invasive and painful procedure; therefore, authors have investigated the need for alternative sources such as dental pulp [3] together with fetal placental membranes [4] and adipose tissue [5,6]. A significant quantity of hMSCs, a non-invasive collection mechanism and an ensured hMSC population that is viable and differentiative are indeed necessary. In light of this, recent investigations suggest that adipose tissue is an ideal source of multipotent adipose-derived stem cells (hASCs) [7,8]. Subcutaneous fat deposits are present in the human body in great quantities and the enzymatic digestion of lipoaspirates will readily isolate ASCs. Liposuction surgeries are widely performed in the US (over 300,000) [9] and in Europe (over 200,000), producing from <30 mL to >6 L of lipoaspirated tissue, which is regularly eliminated with the exception of the Coleman procedure and other procedures that reprocess the tissue [10,11,12]. ASCs are phenotypically similar to hMSCs and also have identical gene expression characteristics. The bone marrow supplies them, and they possess a long culture period. In addition, ASCs represent a valuable therapeutic alternative for various conditions according to in vitro and in vivo studies [13,14,15]. Other studies regarding cell isolation from the adipose tissue of humans and other species suggest that ASCs possess multipotent qualities such as adipocyte, chondrocyte, and osteoblast pathways [16,17,18,19] but also hepatocyte, neuronal-like and pancreatic pathways [20,21,22]. Moreover, ASCs can also be committed to both endothelial and striated and smooth muscle cell lineages [23,24,25]. To date, there is also a lack of clinical reports involving ASCs in cell therapy on humans: the literature mostly reports basic-research-derived protocols and/or other procedures rather than clinical applications [26,27]. Hence, there is a need for a standardised method in a clinical setting which will optimize and unify the process schedule and isolation procedure, as well as the entire tissue manipulation [28,29]. The extensive use and manipulation of stem cells within a clinical setting has been hindered by the Good Manufacturing Practice regulations regarding “cell manufacturing” [30,31], which are not applicable according to the European Parliament and Council (EC regulation no. 1394/2007) as regards minimal manipulation. Nevertheless, in Europe, these protocols will need to meet the requirements recently set by the European Medicines Agency (EMA). The EMA is a decentralized section of the European Union (EU), operating in London since 1995. The Agency is required to assess scientific procedures and to ensure safety measures of treatments and cellular therapy in the European Union. Adequate and accessible procedures are therefore required to obtain autologous hASCs with minimal manipulation for use in clinical settings.

Our study examines an alternative procedure that involves an enzyme-free technique to obtain immediately injectable micro grafts, characterized by a high regenerative potential and non-expanded adipose tissue containing hASCs. The procedure is applicable to a moderately mechanically reduced cluster size of tissue which has been fully immersed—an adipose micro-graft—defining a closed system without enzymes. We herein report the procedure in detail, comparing the phenotype of the system product with methods that involve enzymes. In particular, a non-enzymatic system, named Rigenera (HBW, Turin, Italy), has been designed to collect and prepare human disaggregated biological tissue such as dental pulp, dermis, bone, cartilage, and adipose tissue for re-injection. Rigenera^®^ is a disposable tool that gradually decreases the dimension of the adipose tissue scraps (from spheroidal clusters with a diameter of 1–3.5 mm to smaller ones of 0.2–0.8 mm) and provides a cellular suspension injectable with a needle of small diameter, also called a micro graft. The cell suspension preserves the viability of the regenerative units and of all cells isolated after processing. The complete procedure requires two steps: a surgical step using liposuction and a mechanical step using a disaggregation protocol to obtain the adipose micro-grafts.

## 2. Results

**Figure 1:**
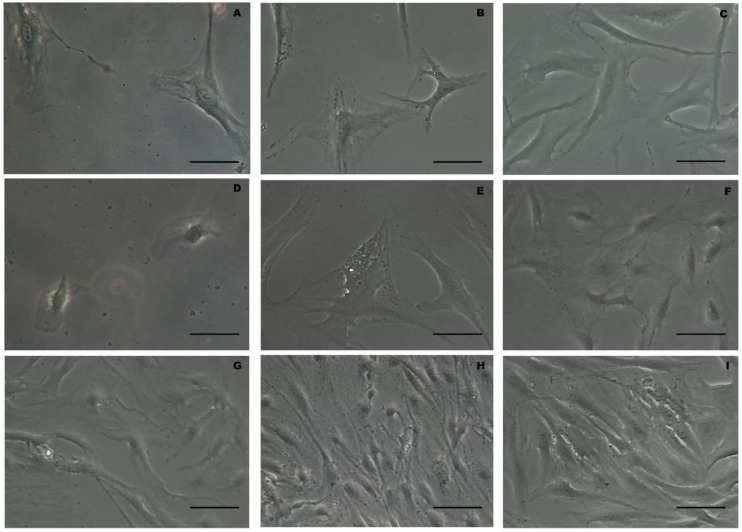
Panel (**A**–**C**) morphological images of Rigenera^®^ methods at 0 h (**A**), 72 h (**B**) and 10 days (**C**) after 30 s of treatment (scale bar 20 μm). Panel (**D**–**F**) morphological images of Rigenera^®^ methods at 0 h (**D**), 72 h (**E**) and 10 days (**F**) after 45 s of treatment (scale bar 20 μm). Panel G-I morphological images of enzymatic digestion methods at 0 h (**G**), 72 h (**H**) and 10 days (**I**) (scale bar 20 μm).

**Figure 2:**
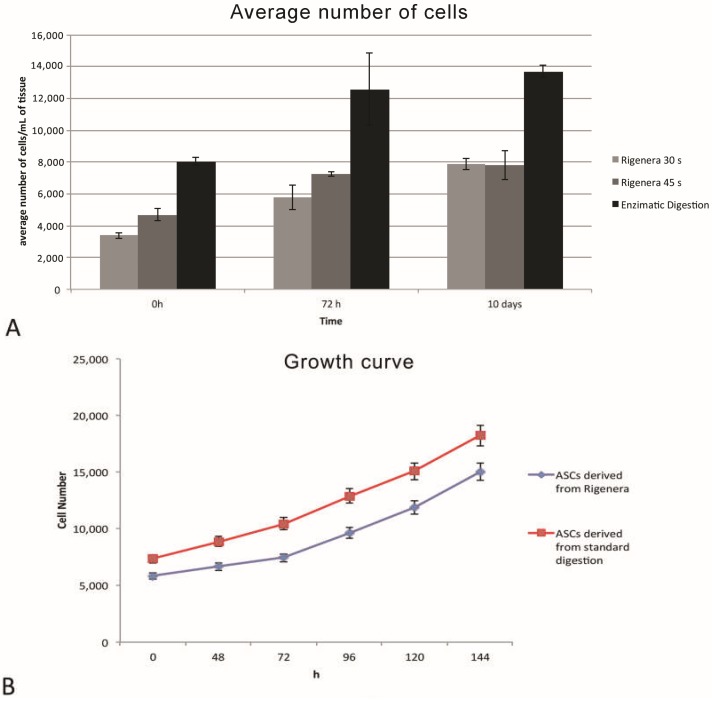
(**A**) Cell viability testing by trypan blue shows the average number of cells in the Rigenera^®^ methods and the enzymatic method at 0 h, 72 h and 10 days. (**B**) Adipose stem cells (ASCs) obtained from the enzymatic method were able to proliferate at a higher rate with a mean doubling time of 96 h and divided at the same speed as the cells isolated with non-enzymatic method.

The cellular elements isolated with the Rigenera^®^ method, shown in Figure 1, have been characterized by their elongated shape, with preserved membranes and a central nucleus. In some cells, small lipid droplets are also detectable. Moreover, there are no substantial morphological differences between the two treatment methods, nor among the two different timings of processing with Rigenera^®^ (30 and 45 s). A cell viability assay, performed with trypan blue staining, shows no significant difference between the Rigenera^®^ and the enzymatic method, as shown in Figure 2A. Concerning the number of isolated cells obtained with Rigenera^®^ and with collagenase, it is possible to observe a higher number of cellular elements with enzymatic digestion of adipose tissue, while, among the two different timings of processing with Rigenera^®^, there are no differences, as shown in Figure 2A. In addition, the cells obtained using the Rigenera^®^ method were characterized by a lower mitosis rate than the population doubling isolated with collagenase.

From the cell count, there is a slight difference between the Rigenera^®^ methods and the enzymatic method in favor of the latter but there are no noticeable differences compared to the two different times used for the centrifugation of adipose tissue in Rigenera^®^ method, as shown in Figure 2B. In addition, the ASCs obtained from Rigenera^®^ method were able to proliferate at a higher rate with a mean Doubling Time of 96 h. The cells isolated with the Rigenera^®^ method are divided at the same speed of the cells isolated with enzymatic method.

**Figure 3:**
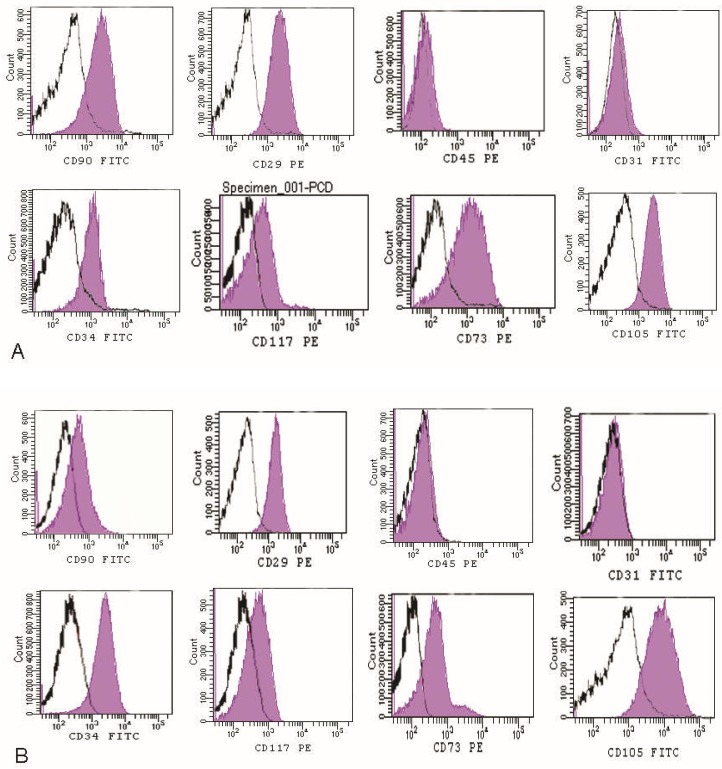
Flow cytometry analysis of the non-enzymatic method (**A**) detected the presence of a cell population that we had previously identified [17,23,32]: including CD34 (35%), CD73 (60%), CD105 (70%) CD90 (70%), CD117 (29%), CD29 (78%), while the cells are negative for CD31, CD45, compared to enzymatic digestion (**B**).

Then, flow cytometry analysis reported a high positivity for MSCs markers, as previously demonstrated [23], including CD34 (35%), CD73 (60%), CD105 (70%) CD90 (70%), CD117 (29%), CD29 (78%), while the cells are negative for CD31, CD45 (hematopoietic markers) (Figure 3) compared to enzymatic digestion.

**Figure 4:**
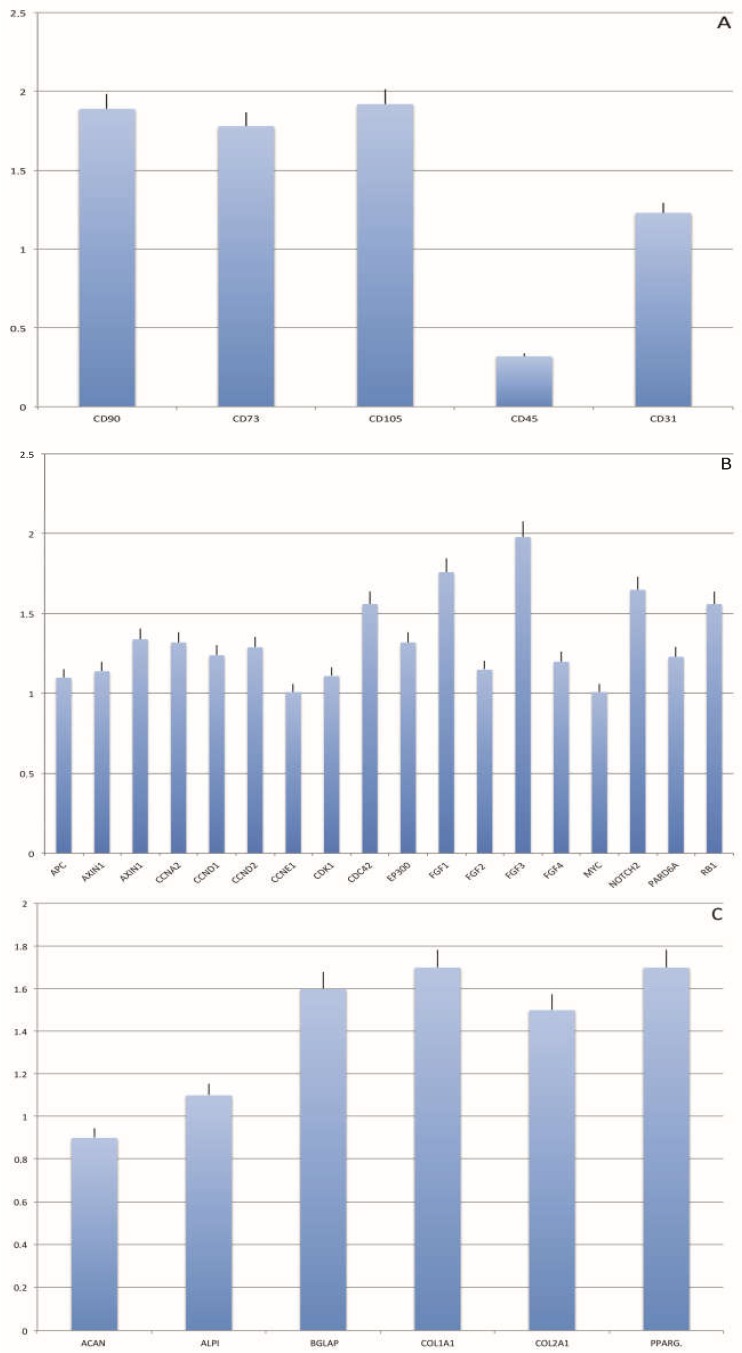
Gene expression of stemness markers (**A**,**B**) and differentiation markers (**C**) in the two digestion methods. Gene expression profile of mesenchymal stem cell specific markers and differentiation-specific markers of non-enzymatic fat disaggregation are reported as ratios (*R*) with respect to the mRNA expression of enzymatic fat digestion [13,32].

In order to confirm the stemness profile of isolated cells, the mRNA of CD90, CD73, CD105, CD45 and CD34 were extracted and measured by real-time PCR. The mRNA of stemness markers, reported in Figure 4, is detectable in all samples. Enzymatic fat digestion was used as control. A significantly comparable mRNA expression for all the markers related to stemness was detected in the non-enzymatic disaggregation method compared to enzymatic digestion, confirming the distribution of stem cells in the fat subsequent to digestion (Figure 4A–C).

## 3. Discussion

European regulations—Directives 2004/23/EC and 1394/2007 established by the European Medicines Agency (EMA)—classify MSCs as advanced medicinal products (ATMPs). These directives state that, in autologous SVF procedures, cell administration is to be performed within the same surgical intervention considering the functionality of the cells to be identical to the fat tissue of the donor. Cellular treatments are not classified as advanced therapy medicinal products. Nevertheless, the EMA defines SVF as an advanced therapy medicinal product in non-homologous practice, specifically involving injured tissue repair in wounds that do not heal, such as scarred tissue and when SVF is used in combination with other products or similar types of MSC cells (http://www.ema.europa.eu/ema; accessed on 26 April 2016 (EMA/298458/2016 corr)). Good surgical practice using manipulated cells as “concurrent treatment” is not defined in EU legislation. Regulation 1394/2007 was established to ensure that patients are not placed at undue risk and that products without proven safety and efficacy are not to be used to treat patients. 

The “minimal manipulation” of the cells is a main objective when attempting to isolate a cell population, and mechanical procedures were set for this so as to avoid regulatory restrictions established by the Food and Drug Administration (FDA) in the US and worldwide. The FDA indeed considers enzymatic procedures in cell populations to be “more than minimally manipulated” and have set heavy restrictions on them, while non-enzymatic methods are considered to be “minimally manipulated” by the FDA. Moreover, procedures that modify the biological, physiological or structural traits of cells or tissues is defined as substantial manipulation. Tissue dissociation to a single cell state usually requires several steps including treatment with collagenase, used to digest the extracellular matrix, and proteases that are broad-specific such as trypsin, used for the dispersion of closely associated cells. In addition, enzyme-digested tissues might also induce cleavage of a wide variety of cell membrane receptors, leading to the alteration of cell biological activities. In summary, the use of collagenase for the separation of cells from the extracellular matrix of tissue is considered a substantial manipulation.

The Rigenera method provides a minimally manipulated derivative of fat tissue obtained via a rapid, safe and easy procedure that enables autologous injection into the donor subject. Besides this, cell expansion or manipulation is avoided, and this is therefore not restricted by the cGMP guidelines. 

The Rigenera product displayed a vascular/stromal arrangement that was well-preserved with perivascular integrity. We evaluated, for each (enzymatic and non-enzymatic) procedure, the SVF yield and correlated it with in vitro outcomes. Data from the flow cytometry analysis of SVF revealed the expression of specific mesenchymal stromal stem cell markers (CD34, CD105, CD90, CD73, CD117), according to the literature [33,34,35]. The percentages of positive cell populations were similar in all experimental groups. Thus, SVF cellular composition was similar within the procedures; the only difference being the cell yield. We particularly observed that the non-enzymatic digestion product was easily transplantable as a fat tissue derivative containing a multitude of hMSCs and few hematopoietic characteristics. When the adipose tissue treated by non-enzymatic digestion underwent tissue culture, an essentially pure population of hMSCs was generated with similar traits to the hMSCs that were isolated from other origins, possessing, for example, a typical adipogenic lineage. Moreover, these results are correlated with an increased expression of mesenchymal stem cells markers such as CD90, CD34, CD73, CD105 that are overexpressed, confirming cytometric analyses, but also a high expression of differentiation markers, indicating the significant multipotency properties of the cells with non-enzymatic disaggregation. In fact, the hASCs originating from Rigenera maintained gene expression including stemness potential, and, besides this, the non-enzymatic digestion method processed lipoaspirates which avoided the use of collagenase and other enzymes, thus contributing to a more adequate preservation of the cell surface and glycocalyx arrangement compared to enzymatic procedures. 

## 4. Materials and Methods

### 4.1. Surgical Procedure to Harvest Adipose Tissue

Adipose tissue harvesting was performed on four women subjected to liposuction for aesthetic purposes, with ages ranging between 28–50 years. Informed consents were obtained prior to tissue collection, according to the ethical guidelines set by the review board for human studies of AOU “Ospedali Riuniti”, Ancona, Italy (Micro-adipose graft_01, 18 May 2017).

For the liposuction, the BEAULI protocol, described by Ueberreiter et al. [36], was followed. In particular, a pulsating water jet was used to infiltrate with contemporaneous aspiration. As for infiltration, the ranges were 1 to 3 from 90 mL/min ± 15% to 130 mL/ min ± 15%. The Klein’s tumescence solution was used, prewarmed to 37 to 38 °C. The cannulas used measured 38 mm in external diameter and were sharp-tipped. Aspiration was performed immediately after the first infiltration. Negative pressure for the aspiration was set to 500 mbar and the Rigenera protocol [37,38] was followed. The obtained lipoaspirate tissue was washed twice with sterile saline solution and prepared for enzymatic or for mechanic digestion.

### 4.2. Isolation, Expansion and Viability of ASCs

A first portion of adipose tissue samples, subsequently processed with Rigenera^®^ technology, were placed in the sterile 16 mL capsules. 12 mL of lipoaspirate and 4 mL of complete culture medium Dulbecco minimum essential medium (DMEM) with 10% of fetal bovine serum (FBS), 1% of a mix of penicillin/streptomycin 1:1 and 0.6% amphotericin B were prepared for mechanic desegregation. Desegregation was performed for 30 and 45 s, in order to determine which of the two timings was most suitable to obtain a better vascular stromal fraction, without affecting its vitality. The second portion of lipoaspirates was digested with collagenase following previously published protocol [32]. Briefly, the lipoaspirates were digested at 37 °C in HBSS with 1 mg/mL collagenase type I (GIBCO life technology) and 2% bovine serum albumin (BSA) for 45 min at 37 °C. After digestion enzyme activity was neutralized with complete medium and centrifuged at 1200× *g* for 10 min, to obtain a high-density pellet, which constitutes the stromal vascular fraction (SVF). It was then resuspended in 5 mL of 160 mM NH_4_Cl and incubated at room temperature for 10 min to lyse contaminating red blood cells. The SVF was collected by centrifugation and filtered through a 70-μm nylon mesh to remove cell debris. SVF of adipose tissue processed with both methods was transferred in 25-cm^2^ flasks (BD Falcon™, Becton Dickinson, Milan, Italy), after addiction of 6 mL of complete medium and incubated at 37 °C in humidified air with 5% CO_2._ The medium was purchased by Sigma-Aldrich (Milan, Italy), while the serum and antibiotic mix were acquired by GIBCO Life Technologies (Waltham, MA, USA). When at confluence, cells were treated with trypsin-EDTA 1% (GIBCO Life Technologies, USA), harvested and centrifuged at 1200 rpm for 5 min. The supernatant was discarded and cells pellet was resuspended in 10 mL of complete medium, placed in 75 cm^2^ plates and incubated at 37 °C and 5% of CO_2_ until 80% confluence was detectable. Cell viability was evaluated by trypan blue staining at 0 h, 72 h and 10 days.

### 4.3. Growth Curve

Both the cells excreted with Rigenera^®^ and enzymatic digest were plated in 6-well plates. Cells were collected and re-suspended in PBS for a period of 96 h at a 12-h interval. Cell suspension was counted under a microscope at 20× magnification and viable cells were counted and presented on a linear graph. The doubling time (DT) was set from the growth curves or via the following formula:

DT = (*t* − *t*_0_)log2/(log*N* − log*N*_0_) (1)

where *t* (time) and *t*_0_ represented when the cells were counted, and *N* and *N*_0_ represented the cell numbers at times *t* and *t*_0_, respectively.

### 4.4. Flow Cytometry Analysis and Phenotype Characterization

At confluence, the cells derived from the Rigenera digestion method or the lipoaspirates from the digestive solution were detached with trypsin-EDTA (200 mg/L EDTA, 500 mg/L trypsin; Cambrex, Milan, Italy). At least 200,000 cells were, directly placed in incubation using fluorescent conjugated antibodies for 30 min at 4 °C then washed and re-suspended in 0.6 mL. Specimens were examined using FACS Aria II flow cytometry (Becton & Dickinson, Mountain View, CA, USA). The antibodies herein investigated were: anti-CD117 PE (c-kit) (Miltenyi-Biotech, Calderara di Reno, Bologna, Italy); anti-CD34 FITC and PE (Miltenyi-Biotech); anti-CD90 FITC (BD Pharmingen, Buccinasco, Milano, Italy); anti-CD105 FITC (Santa Cruz, CA, USA); anti-CD73 PE (Miltenyi-Biotech); anti-CD29 PE (Miltenyi-Biotech); anti-CD31 FITC (Miltenyi-Biotech); and anti-CD45 Cy and PE (BD Pharmingen).

### 4.5. Real-Time PCR

Real-time PCRs were carried out using the designed primers at a concentration of 300 nM and FastStart SYBR Green Master (Roche, Monza, Italy) on a Rotor-Gene 3000 (Corbett Research, Sydney, Australia). Real-time PCR was also performed according to the user’s manual for the human mesenchymal stem cell Profiler PCR Array (SABiosciences, Frederick, MD, USA) and using RT2 SYBR Green ROX FAST Master Mix (Qiagen, Milan, Italy). The data were analyzed using Excel-based PCR Array Data Analysis Templates (SABiosciences). The thermal cycling conditions were as follows: 15 min denaturation at 95 °C, followed by 40 cycles of 15 s denaturation at 95 °C, annealing for 30 s at 60 °C, and 20 s elongation at 72 °C. Differences in gene expression were evaluated by the 2ΔΔ*C*_t_ method. Values were normalized to the expression of glyceraldehyde-3-phosphate dehydrogenase (GAPDH) internal reference whose abundance did not change under our experimental conditions. Experiments were performed with three different cell preparations and repeated at least three times.

The results are reported as ratios with respect to the mRNA expression of enzymatic fat digestion.

### 4.6. Statistical Analysis

All data were statistically analyzed using a one-way ANOVA test. The threshold for statistical significance was set at *p*-values < 0.05. Repeatability represented as a standard deviation to calculate the differences between measurements using SPSS 16.0 software (SPSS Inc., Chicago, IL, USA) for assessment.

## 5. Conclusions

Adipose tissue has recently been considered in relation to plastic surgery and regenerative treatments, with successful investigations into isolated SVF and ASCs. However, the cellular treatment methods have not been fully exploited, especially due to restrictive worldwide measures, which fundamentally entail a common operating protocol, toxin- and xeno-free reagents, replacing enzymes and enabling a rapid monitoring of quality standards to ensure an adequate cell identity and the efficiency of the donor tissue. The objective of a procedure that enables cell isolation from adipose tissue has been set: a procedure that ensures a sterile and safe environment and cell material stability but not all cell isolation systems are closed, and where cleanroom systems are required for a sterile, isolated setting. 

Enzymatic digestion is currently the gold standard procedure, providing more adipose-derived stromal cells from adipose tissue; however, we demonstrate that our mechanical methods enable the easy extraction of good quality adipose-derived stromal cells, which could be sufficient for routine clinical use after clinical qualification. In addition, mechanical methods allow the isolation of adipose-derived stromal cells with stemness and immunosuppressive properties similar to those obtained after collagenase digestion. Our processing protocol is rapid, simple, and reproducible, providing adipose-derived stromal cell–enriched stromal vascular fraction.

Our study displays a unique product to use autologously, obtained from enzymatic digestion, that provides an efficient alternative in the treatment of damaged tissue thanks to its minimal manipulation factor and its clinical accessibility. Finally, laboratory and pre- clinical studies will be required to confirm the safety of stromal vascular fraction and to ensure a strict clinical framework for its use in regenerative medicine.
